# Adaptation, perceptual learning, and plasticity of brain functions

**DOI:** 10.1007/s00417-016-3580-y

**Published:** 2017-01-14

**Authors:** Jonathan C. Horton, Manfred Fahle, Theo Mulder, Susanne Trauzettel-Klosinski

**Affiliations:** 1 Beckman Vision Center, University of California, San Francisco, USA; 2 Center for Cognitive Sciences, University of Bremen, Bremen, Germany; 3 Royal Netherlands Academy of Arts and Sciences, Amsterdam, The Netherlands; 4 Vision Rehabilitation Research Unit, Center for Ophthalmology, University of Tübingen, Tübingen, Germany

**Keywords:** Brain plasticity, Adaptation, Perceptual learning, Visual cortex, Motor cortex, Rehabilitation

## Abstract

The capacity for functional restitution after brain damage is quite different in the sensory and motor systems. This series of presentations highlights the potential for adaptation, plasticity, and perceptual learning from an interdisciplinary perspective. The chances for restitution in the primary visual cortex are limited. Some patterns of visual field loss and recovery after stroke are common, whereas others are impossible, which can be explained by the arrangement and plasticity of the cortical map. On the other hand, compensatory mechanisms are effective, can occur spontaneously, and can be enhanced by training. In contrast to the human visual system, the motor system is highly flexible. This is based on special relationships between perception and action and between cognition and action. In addition, the healthy adult brain can learn new functions, e.g. increasing resolution above the retinal one. The significance of these studies for rehabilitation after brain damage will be discussed.

## Introduction by S. Trauzettel-Klosinski

This symposium highlighted the potential for learning and re-learning after visual and motor cortex lesions in the adult brain from an interdisciplinary perspective. We considered mechanisms such as adaptation, plasticity, and perceptual learning of different brain functions, as well as their applications for rehabilitation in patients with brain damage. Additionally, the potential for visual learning in the normal human brain was demonstrated.

In the visual system, the potential for recovery in the primary visual cortex is limited (part 1 by Jonathan Horton). Visual field defects caused by embolic stroke are constrained by the organization of the blood supply of the occipital lobe with respect to the retinotopic map. In terms of the arrangement and plasticity of the cortical map, it will be explained why some patterns of visual field loss and recovery following stroke are common, whereas others are essentially impossible. This is especially true along a visual field strip of constant width along the vertical meridian.

While the restitutive capacities of the primary visual cortex are limited, compensatory mechanisms can be very effective (part 2 by Susanne Trauzettel-Klosinski). They can occur spontaneously and can further be enhanced by training. In hemianopia, for example, fixational eye movements and scanning saccades can shift the visual field border towards the hemianopic side and improve spatial orientation and mobility.

In contrast to the visual system, the human motor system is highly flexible (part 3 by Theo Mulder). It is updated continuously by itself on the basis of sensory input and activity. The plasticity of the motor system is based on a special relationship between perception and action, as well as between cognition and action. New approaches to rehabilitation, for example by motor imagery, give an outlook on future possibilities.

Additionally, the healthy adult brain can learn new visual functions (part 4 by Manfred Fahle), for example the enhancement of resolution, which is higher than that of the retina. These functions, especially hyperacuity, can also be trained.

The authors will present a summary for each of the four talks.

## Part 1: visual field recovery after lesions of the occipital lobe by Jonathan C. Horton

**Fig. 1:**
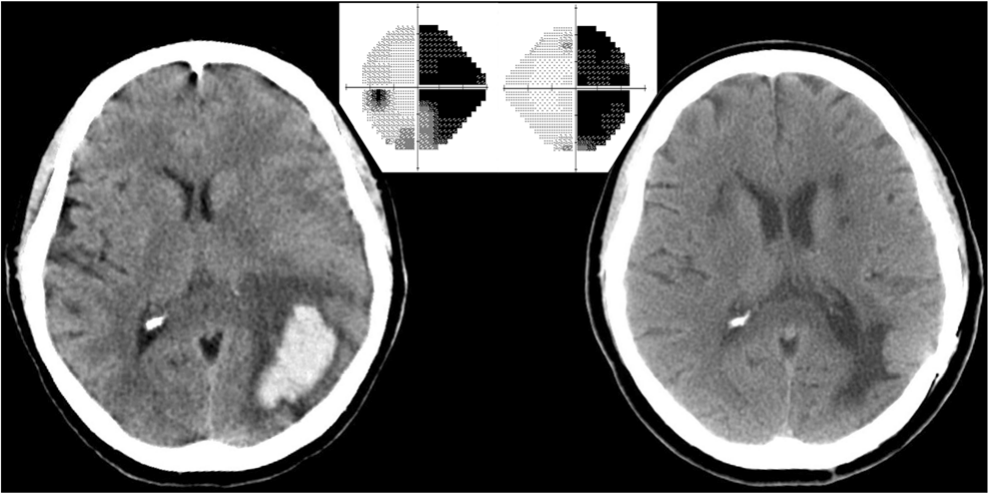
CT scan showing an acute left parietal hematoma, causing a right homonymous hemianopia. A CT scan performed 5 months later shows damage to the left optic radiations. The visual field cut never recovered

Recently, I attended a 60-year-old woman who had a spontaneous left parietal hemorrhage (Fig. 1). She underwent an emergency craniotomy to evacuate the hematoma. Her main deficit was a severe aphasia, which improved slowly. Once she regained sufficient ability to communicate, she complained about her vision on the right side. Her examination showed a total, macula-splitting right homonymous hemianopia. She has made nearly a complete recovery from her stroke, except for this devastating visual field cut. It has made reading a chore, forced her to give up driving, and will prevent her from returning to her job. This is a common scenario: after surviving a neurological disaster, patients discover that vision loss represents their most serious and enduring deficit. Why does central vision loss persist, and remain so stubbornly resistant to treatment?

**Fig. 2:**
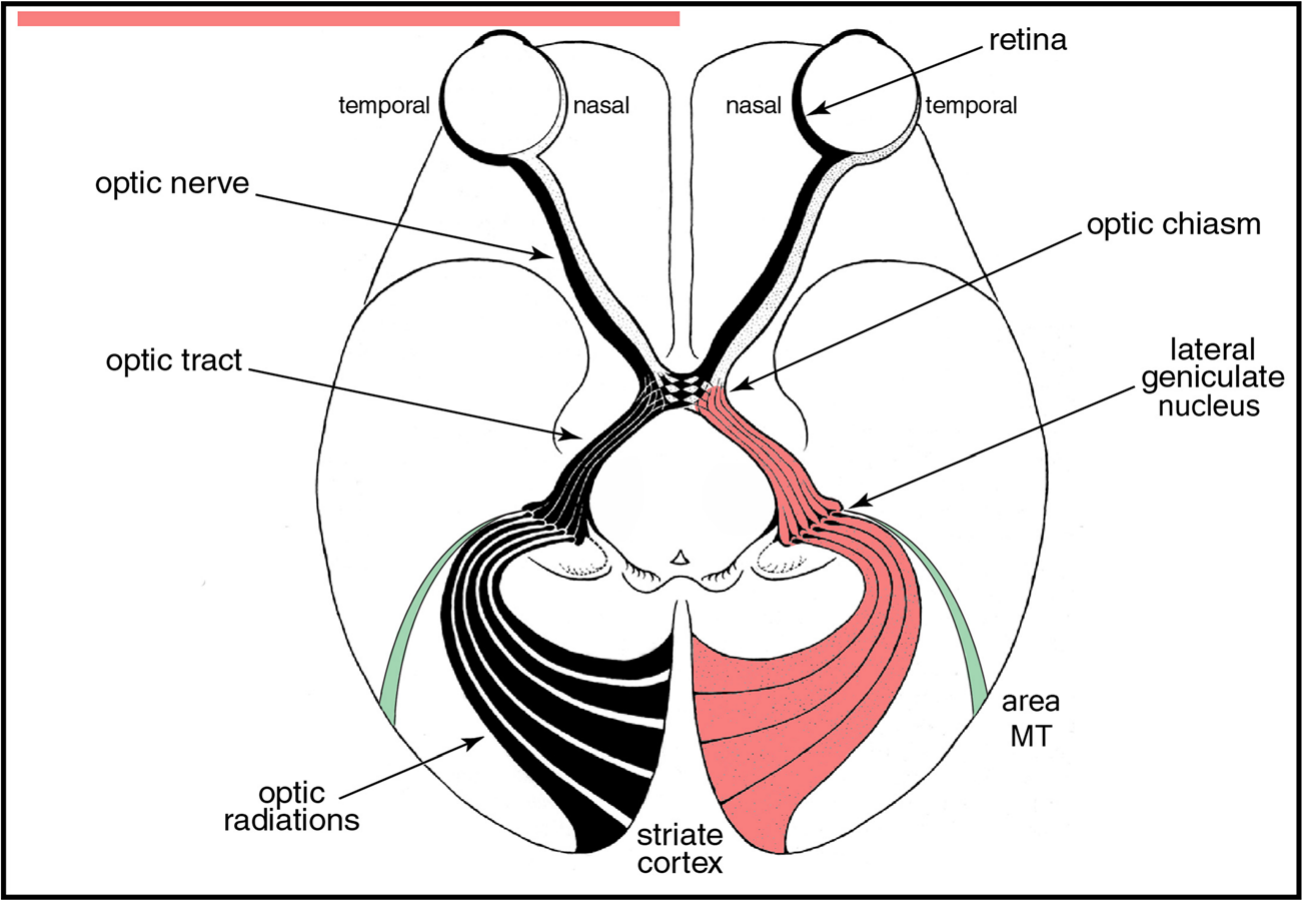
Retinal input is conveyed to the primary (*striate*) cortex by a two-neuron chain, crossing a single relay in the lateral geniculate nucleus. Injury at any point cuts off visual perception, although a small projection (*green shading*) from the lateral geniculate to area MT allows “blindsight” in patients with homonymous hemianopia caused by a post-chiasmal lesion (*pink shading*). After Polyak (1957)

The answer lies in the organization of the visual pathway from eye to cortex. Retinal ganglion cell axons that are responsible for conscious perception project to the lateral geniculate nucleus. It serves as a relay station, boosting the information content of outgoing spikes compared with incoming spikes by integrating and filtering retinal signals [1]. Geniculate neurons send their projection to layer 4 of the primary visual cortex. Simply by crossing a single synapse in the thalamus, retinal output is conveyed directly to the primary visual cortex. In a sense, the retino-geniculo-cortical pathway is the aorta of our visual system (Fig. 2). After initial processing in the primary visual cortex, signals are analyzed in surrounding cortical areas that are specialized for different attributes, allowing us to perceive the images that impinge upon our retinae.

Sprawling across the brain from eyes to occipital lobe, the retino-geniculo-cortical pathway is vulnerable to a multitude of neurological insults. As every ophthalmologist knows, injury to the optic nerve, chiasm, or tract causes retrograde degeneration of ganglion cells in the retina. Downstream from the site of injury, retinal ganglion cell axons undergo anterograde degeneration. Their terminals disintegrate in the lateral geniculate nucleus. At present, there is no way to regenerate lost retinal ganglion cells, and even if there were, there is no way to guide their axons to terminate in the correct location in the lateral geniculate nucleus.

**Fig. 3:**
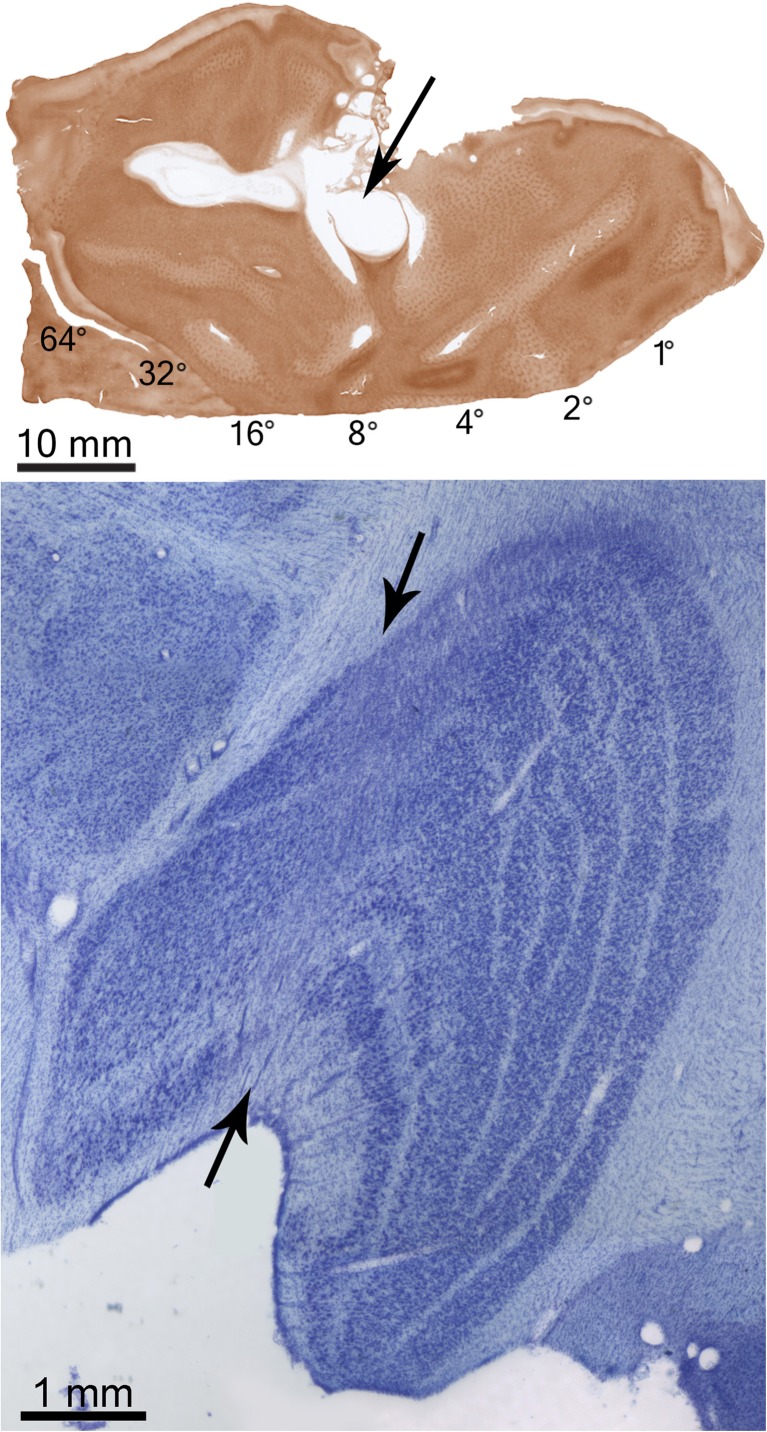
(*Top*) Flattened tissue section reacted for cytochrome oxidase showing a large lesion (*arrow*) of the primary visual cortex in a monkey. (*Bottom*) The lesion produced a swath of cell loss, visible in a Nissl-stained section, running through all layers of the lateral geniculate nucleus (*arrows*). Relay neurons in the lateral geniculate die because their axon terminals are destroyed in the cortex

By the same token, injury to the visual cortex or optic radiations causes retrograde degeneration of neurons in the lateral geniculate nucleus. An example of a lesion in the primary visual cortex of a monkey is shown in Fig. 3. It produced a zone of cell loss running through all the layers of the lateral geniculate nucleus. It is important to bear in mind that a lesion of the calcarine fissure not only destroys cortical neurons, but amputates visual signals emanating from the lateral geniculate nucleus. Even if one could repair the cortical damage, loss of input from the lateral geniculate would be enough to shut down vision.

The exquisite preservation of topographic order in the visual system compounds the functional impairment wreaked by lesions of the retino-geniculo-cortical pathway. Each location in the visual field is represented serially at precise anatomical sites along the pathway, with no redundancy. Once a site is destroyed, vision is cut off, because there is no other way around the choke point. In this respect, the visual system is quite different from the auditory system. VIIIth nerve output is supplied to the dorsal cochlear nucleus, ventral cochlear nucleus, medial accessory nucleus, and superior olivary nucleus on each side of the medulla. From the medulla, auditory signals are fed to the nucleus of the lateral lemniscus and the inferior colliculus, again on both sides of the brainstem. They ultimately reach the temporal lobes via the medial geniculate bodies. The crucial point is that information can reach the auditory cortex via several routes, because there exist multiple decussations and parallel relay streams. Moreover, the cortex in each hemisphere contains a representation of all frequencies and all locations in space. Consequently, no deficit ensues after a unilateral lesion of primary auditory cortex. Clearly, different rules pertain in auditory, visual, motor, and language cortex (see Mulder T, part 3 below).

Years ago, excitement followed reports that topographic maps are plastic in the visual cortex, even in adults [2, 3]. In experimental animals, lesions were made in the retina with a laser, silencing a corresponding zone in the cortex. Afterwards it was observed that the silent cortical zone eventually becomes responsive to stimulation from surrounding, healthy retina. This result was surprising, because it was thought that anatomical connections in the mature cortex lack the capacity to fill in large gaps created by deafferentation. Unfortunately, the phenomenon was not replicable in other laboratories [4, 5]. Even if real, it is hard to see how filling in could benefit visual function. The scotoma from the retinal laser burn remains, regardless of what happens in the cortex.

**Fig. 4:**
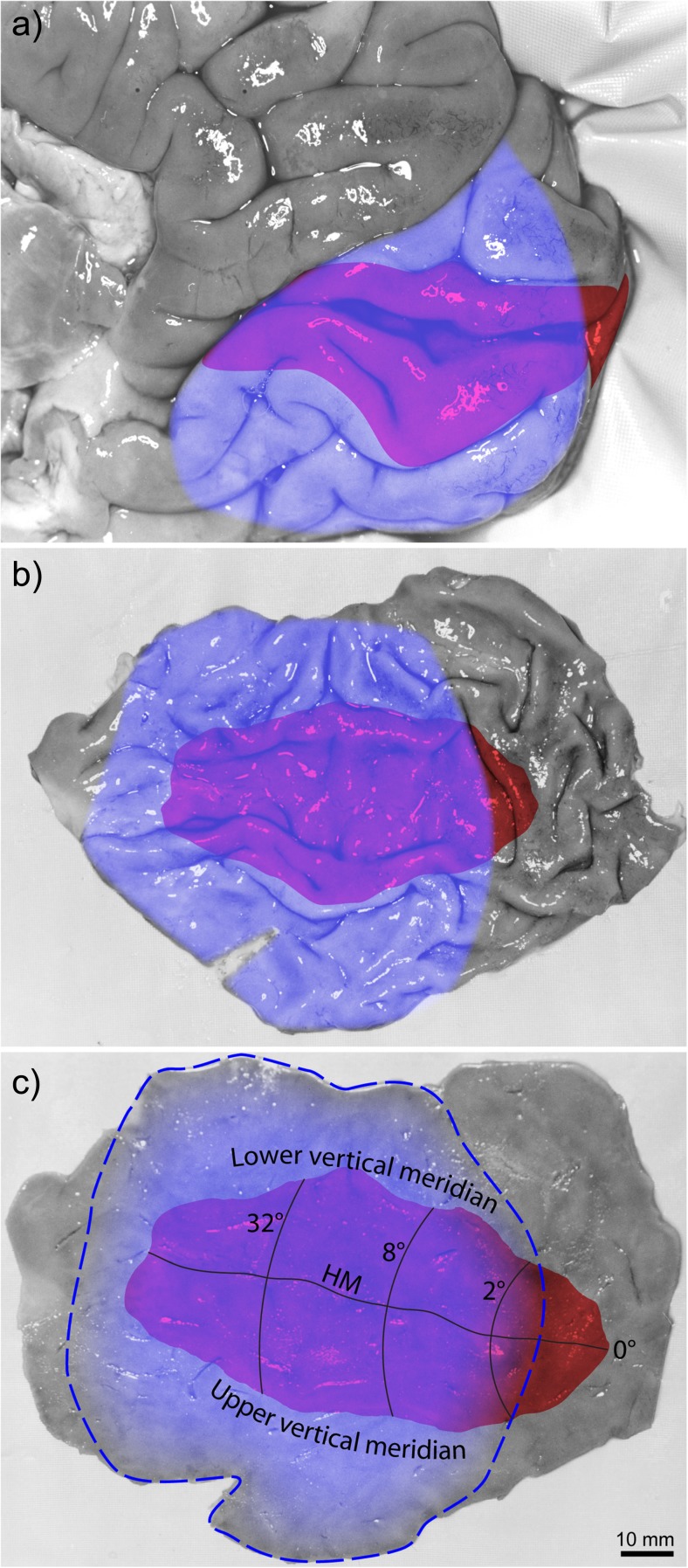
**a** Right occipital lobe, with red shading to indicate the primary visual cortex. A large stroke (*blue shading*) from occlusion of the posterior cerebral artery is shown. **b** The calcarine fissure is opened to reveal the primary visual cortex. The stroke extends even beyond the edge of the semi-flattened cortex, except posteriorly, where cortex is supplied by the middle cerebral artery. **c** Flattened sheet of cortex, marking the boundaries of the stroke in (**b**) with a *dashed line*. Months after stroke, some recovery may occur at the fringes of the infarct, reducing the amount of cortical damage (shown schematically by shrinkage of the *blue shading*). However, the stroke still extends far beyond the borders of the primary visual cortex, so no recovery of visual field along the vertical meridian should be expected

After a stroke, physical therapy can help patients recover motor function. Can vision therapy do the same for the visual system, by shrinking field defects? Sabel and colleagues have described partial recovery of homonymous hemianopia through computer-based rehabilitation therapy [6]. Subjects undergo a daily training regimen, detecting stimuli presented on a computer screen while they maintain fixation. The hope is that stimulation of visual field represented by partially damaged brain tissue at the fringe of a stroke can promote recovery. Data have shown that improvement is particularly apt to occur along the vertical meridian. In the occipital lobe, the vertical meridian corresponds to the perimeter of the primary visual cortex (Fig. 4). Strokes extend far beyond this frontier, but they produce a field cut that respects the vertical meridian. The sharp vertical edge to the hemianopia is because the intact visual hemifield is represented in the other hemisphere of the brain. It is remote from the stroke responsible for the hemianopia. This fact vitiates the theory that visual field recovery along the vertical meridian is due to resuscitation of damaged, but viable cortex at the fringes of the lesion.

After onset of a hemianopia, patients learn to make frequent saccades towards their blind side, perhaps as a compensatory mechanism [7]. This behavior is so powerful that patients have trouble maintaining prolonged fixation on a stationary target. The strip of “recovered” visual field along the vertical meridian occurs because patients sneak frequent glances to the blind side. When testing is done by controlling fixation rigorously during perimetry, no significant benefit can be detected from vision restoration therapy [8]. In other words, field improvement from vision therapy is an artifact of sloppy psychophysical testing.

Even in patients with infarction of calcarine cortex from a posterior cerebral artery occlusion, a crude ability to localize large moving objects is sometimes preserved. This residual visual capacity has been given the catchy name “blindsight” [9]. It may be due to a small projection from the lateral geniculate nucleus to a region in the parietal lobe known as “area MT” [10]. This region was discovered because it stains prominently for myelin, just like the primary visual cortex. It can be thought of as a small, accessory region of primary visual cortex, hanging like Tasmania off the Australian continent. Silencing the projection from the lateral geniculate nucleus to Area MT abolishes blindsight in monkeys [11]. Area MT lies outside the vascular territory of the posterior cerebral artery, so it remains functional after occipital lobe stroke. Nonetheless, blindsight is too weak to provide much help to patients with hemianopia. One must concede that the goal of restoring sight after damage to the retino-geniculo-cortical pathway remains a profound challenge for scientists and clinicians. Ultimately, success will require gaining the ability to regenerate damaged neuronal tissue, learning how to graft it onto the patient’s brain, and then hooking it up properly to allow useful function.

## Part 2: compensatory adaptation to visual field loss after brain damage by Susanne Trauzettel-Klosinski

Hemianopia leads to orientation disorder, indicated by bumping into objects or persons, problems with route finding and impaired communication. In addition, if the visual field defect includes the visual field center, reading is severely impaired. These impairments result in restricted participation in society and a severe reduction of quality of life.

### Spontaneous adaptive mechanisms

For rehabilitation of hemianopia, the investigation of spontaneous adaptive mechanisms is crucial: Are these mechanisms helpful? Which patients have the potential to develop them? Can they be trained?

**Fig. 5:**
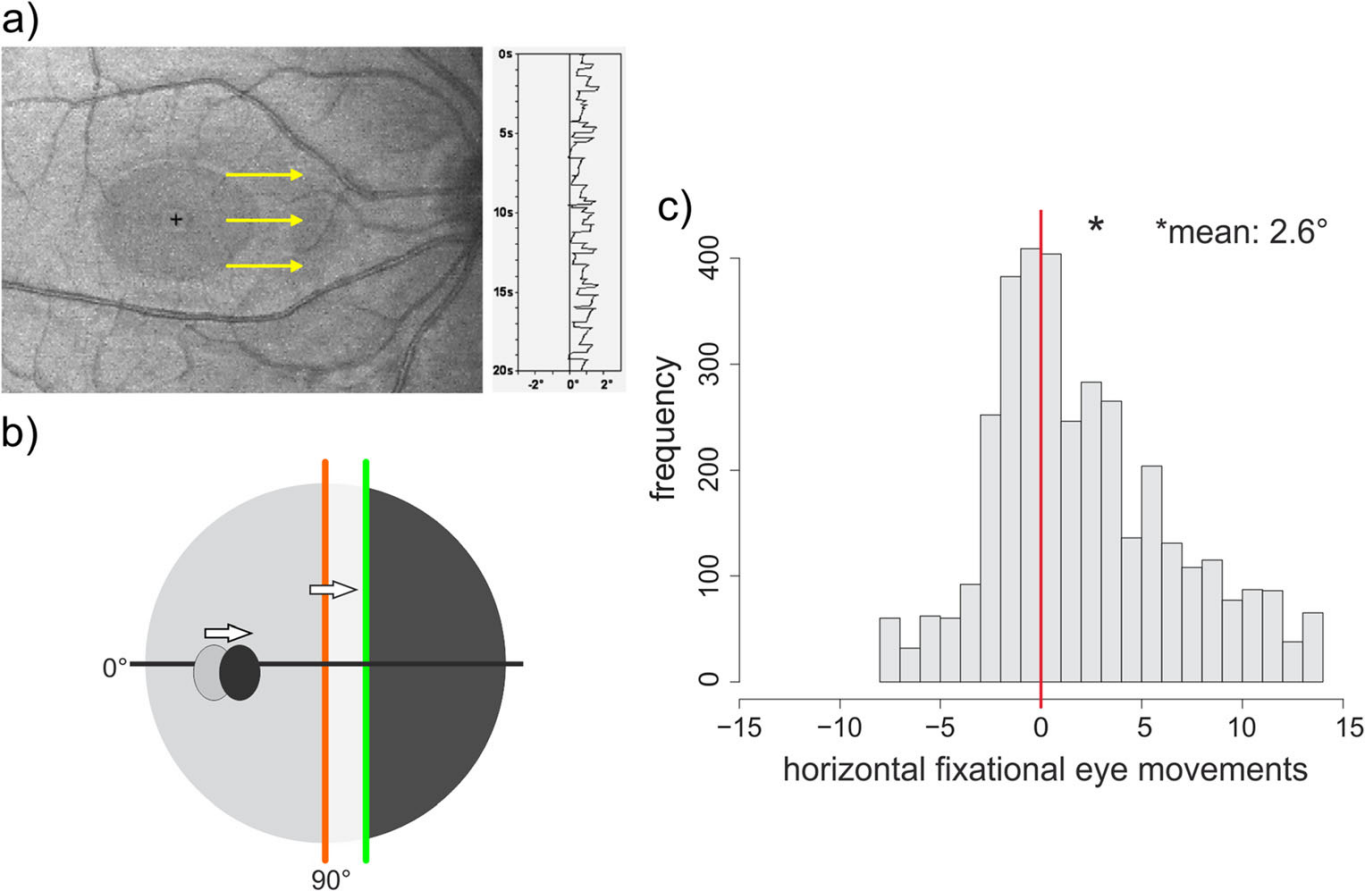
Fixational eye movements during fixation of a cross are asymmetric towards the blind side, shown for right hemianopia: **a** assessment by scanning laser ophthalmoscope (SLO), example of one patient. **b** in conventional perimetry (schematic), the visual field defect and the blind spot are shifted towards the blind side. **c** distribution of fixational eye movements in 25 patients with right hemianopia with absent or small (<4°) macular sparing assessed by SLO (based on 1000 video fields per patient): the mean is shifted to 2.6 degrees to the right and is significantly different from normal distribution modified after [12]

Fixational eye movements occur as a physiological phenomenon in healthy subjects to prevent fading and to maintain constant vision during fixation (for references see [12]). In hemianopia, the fixational eye movements are asymmetric towards the blind side, which causes a shift of the visual field border to the blind side [12, 13]. This shift of the vertical field border can be misinterpreted as an enlargement of the visual field (Fig. 5).

Scanning eye movements: While viewing naturalistic scenes, patients were described to show increasingly different fixation patterns from normal subjects, which indicates a compensating strategy [14].

Asymmetric eye movements towards the hemianopic side, which are small during fixation, occur as larger saccades to scan the blind hemifield by using the full field of gaze, i.e. to enlarge their “functional visual field” (for details see [15, 16]).

Regarding saccadic accuracy, short-term adaptation has been described [7], but insufficient long-term adaptation [12], which is indicated by the increased number of dysmetric saccades during gaze shift to the blind side.

Furthermore, a shift of attention to the blind side can be helpful to promote scanning saccades, because they are preceded by movements of attention. A head turn alone does not change the visual fields. However, a head turn in combination with scanning eye movements leads to an extension of the functional visual field by using the full field of gaze.

Exotropia with anomalous retinal correspondence can extend the binocular visual field, which is then a contraindication for strabismus surgery [17].

### Rehabilitation of the hemianopic orientation disorder

For intervention studies the following general aspects have to be considered:

1. Specificity:
  spontaneous recovery has to be excluded
  a placebo effect has to be ruled out by use of a control group
2. Quality of testing methods for assessing the effect :
  objectivity
  validity (e.g. can the test show causal connections?)
  reliability (e.g. exactness, repeatability)
3. Aim of the intervention
  Is the effect clinically relevant?
  Is the effect persistent after training?

The main approaches in recent years were substitutive, restitutive, and compensatory.

Literature research regarding rehabilitation in hemianopia was performed using Cochrane Reviews and randomized controlled trials (RCTs) in Cochrane and Pubmed for the period 1990 – April 2016. The reference list of part 2 is restricted mainly to overview articles and RCTs. Those after 2010 are cited directly in the list below, the majority of those published before 2010 are listed in the overview articles [15, 16].

#### The substitutive approach

The use of peripheral prisms to expand the functional visual field without central diplopia yielded positive subjective reports by the patients, but a conclusive judgment of the benefit is not possible at present.

#### The restitutive approach

The aim of restitutive training is to enlarge the visual fields by reactivating incompletely damaged neurons in the blind hemifield by visual stimulation. Earlier studies using visual stimulation along the vertical border of the field defect reported visual field enlargement [6], but it was later shown by fundus-controlled perimetry that fixational eye movements shift the field defect towards the hemianopic side, which can be misinterpreted as an enlargement of the visual field [8, 18]. At present, there are no evidence-based studies available that show an effect of training to restitute the visual field. (Cochrane review [19]; for a recent review see [20] and Horton Part 1 in this article).

Regeneration of neurons in the primary visual cortex (V1) should be distinguished from extrastriate activation, also called the “blind-sight phenomenon” (see Horton part 1 above). “Phylogenetically old” pathways via the lateral geniculate nucleus that bypass area V1, can be partly re-activated by intense training. In some patients, this can lead to mostly unconscious perception [20]. It is still an open question, whether residual vision of this kind can be improved to a level that is relevant to daily life.

#### The compensatory approach

The spontaneous mechanism of generating scanning eye movements towards the hemianopic side is used and enhanced by compensatory saccadic training. Earlier non-controlled studies reported positive effects, but the specificity of the method was not proven. The specific positive effects of explorative saccadic training was proven in the first randomized and controlled trial by our group [21]: It selectively improved saccadic behavior, performance in an everyday search task (searching objects on a table) and natural scene exploration. The effects were also present in patients with longstanding hemianopia. The new saccadic strategy could be applied to everyday life and the training effect remained stable after the end of the training. Quality of life in the social domain improved.

**Fig. 6:**
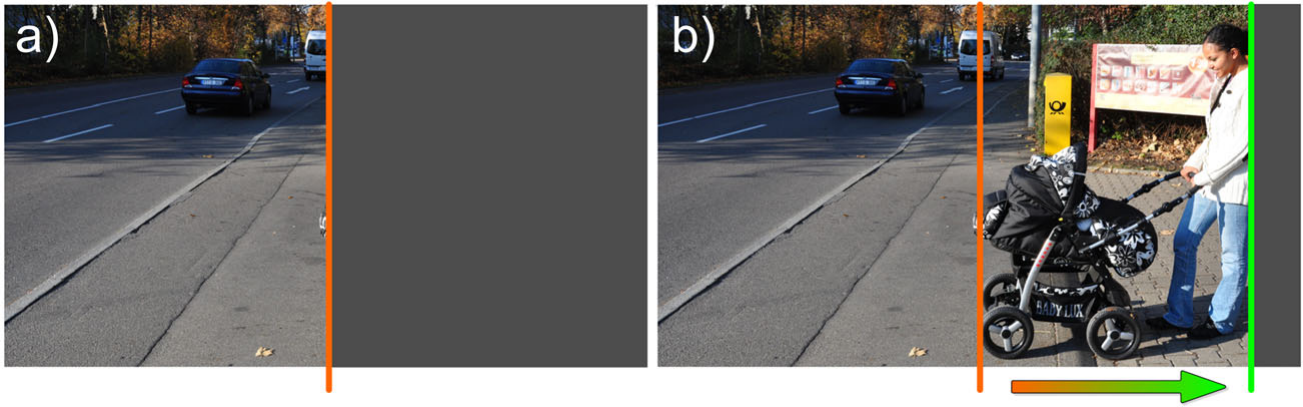
Exploration of a natural scene in right hemianopia: a) without eye movements, b) with scanning eye movements the field of gaze is utilized and obstacles, here the baby stroller, can be seen in time

Figure 6 shows the functional visual field for a hemianopic patient viewing the scene without eye movements (a) and with scanning eye movements (b). The detection of obstacles, here the baby stroller, is especially valuable for avoiding collisions.

In the meantime, several randomized controlled trials (RCTs) have been performed (for an overview see [15, 16]) that showed improvement of exploration and orientation by ways of audio-visual stimulation, attentional training, a combined reading and exploration training [22], and a purely horizontal saccadic training task [23]. A systematic review on multisensory stimulation did not allow a valid conclusion about the effectiveness of this intervention [24]. Another interesting approach was reported using anti-saccadic training [25].

In summary, it is evident that after brain damage regeneration of the occipital cortex is quite limited, whereas compensatory plasticity by extrastriate activation can lead to changes in gaze strategy with an improved adaptation to the demands of everyday life.

### The hemianopic reading disorder

**Fig. 7:**
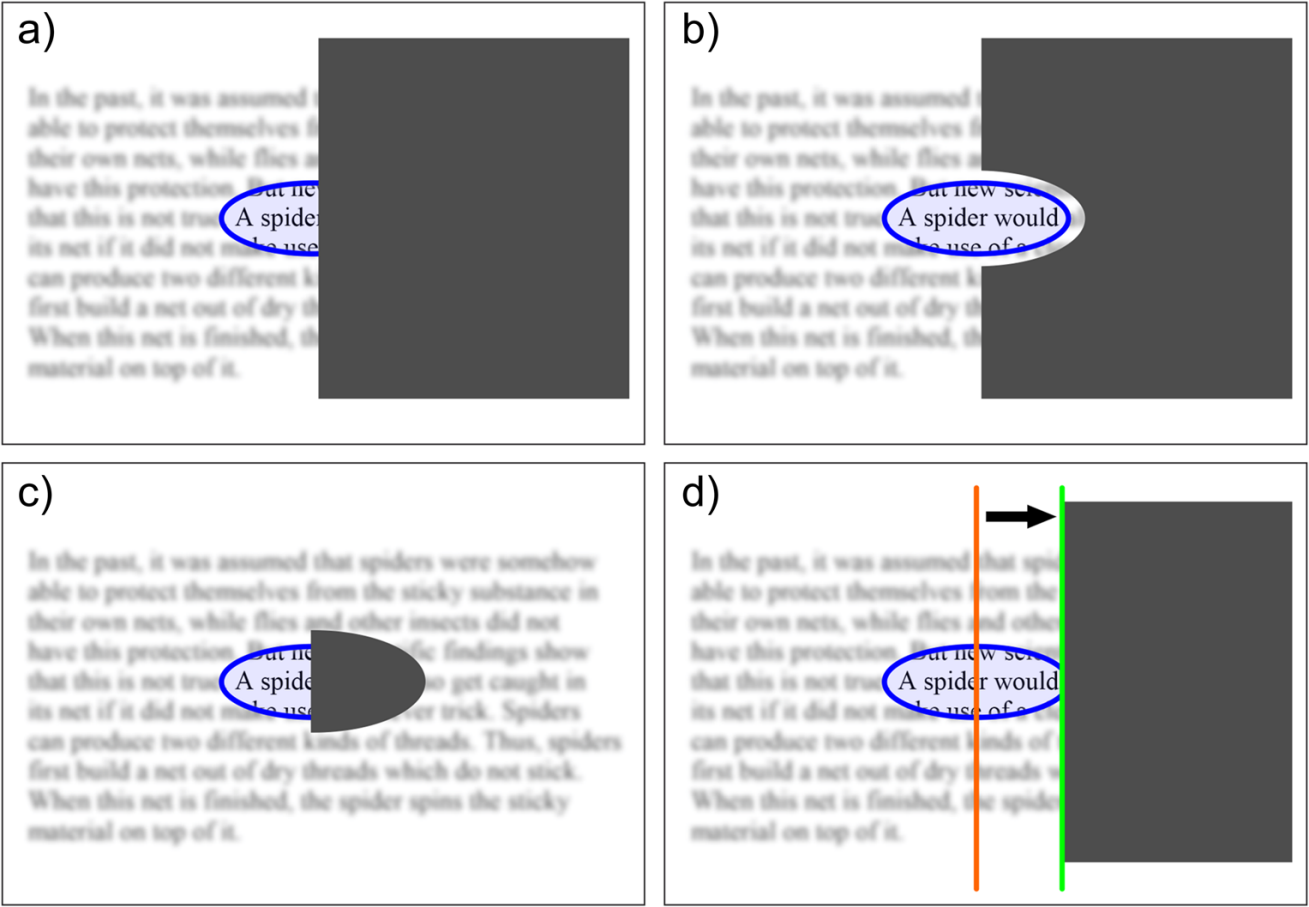
Reading in hemianopia depends on the configuration of the field defect and the available perceptual span during one fixation: **a** In macular splitting, half of the reading visual field is covered and functionless, resulting in severe reading impairment. **b** In macular sparing, the reading visual field can be spared and reading can be normal. **c** A paracentral homonymous scotoma leads to severe reading impairment. **d** Eccentric fixation shifts the field defect towards the hemianopic side and creates a small perceptual strip along the vertical field border, a favorable adaptive mechanism

Reading performance in hemianopia depends highly on its configuration: In macular splitting, half of the reading visual field is covered by the scotoma and is dysfunctional (Fig. 7a). In patients with macular sparing, the reading visual field (perceptual span during one fixation) can be fully spared and reading is not impaired (Fig. 7b). On the other hand, a small paracentral homonymous scotoma can cover half of the reading visual field and lead to severe reading impairment (Fig. 7c).

Furthermore, the reading performance depends on the side of the field defect in regard to the reading direction [15, 16]: In languages that require moving the eyes from left to right along the line, patients are much more impaired by a right hemianopia, indicated by an increased number of saccades and regressions and a severely reduced reading speed. If a left hemianopia is present, patients have the problem of finding the beginning of the next line, indicated by several hypometric saccades during the return sweep.

#### Spontaneous adaptive mechanisms for reading

A promising adaptive mechanism is eccentric fixation: a minority of patients (approximately 20%) are able to use a slightly eccentric fixation locus, which shifts their visual field border towards the hemianopic side and creates a narrow seeing strip along the vertical field border (Fig. 7d). They can use this mechanism by sacrificing a bit of visual acuity and gaining a slightly enlarged reading visual field, which leads to homonymous eccentric fixation [15, 16]. Another favorable adaptive mechanism is making predictive saccades, especially in patients with left hemianopia, who can learn to apply a single hypermetric predictive saccade to find the beginning of the new line.

#### Rehabilitation of the hemianopic reading disorder

Only few RCTs are available: It has been shown that reading scrolled text for right hemianopia was effective to improve reading speed [26]. Furthermore, reading speed increased by performing a search task in a line of words [22] and anti-saccade training [25]. Other approaches, based on clinical experience, are to help orientation on the page by use of visual and tactile aids, for example the index finger, a ruler or a slightly vertically magnifying ruler with a red guideline. Turning the text into a vertical or diagonal orientation has not been studied systematically yet.

To summarize, to aid the rehabilitation of hemianopia, only compensatory methods have been shown to be effective in evidence-based studies to date: For general orientation, by applying visual and audio-visual search tasks, attentional training and saccadic/anti-saccadic tasks. For reading, scrolled text for right hemianopia and search task in a line of words.

## Part 3: brain plasticity and recovery of motor function by Theo Mulder

Human motor behavior is not the result of a series of detailed muscle-specific central commands, but is characterized by an extreme flexibility. Almost without any effort we can pick up a cup with the right hand, with the left hand, we can even pick it up by using our feet as the main effector organs. We can walk forward, backward, we can jump, dance, run, shuffle, and produce all sorts of silly walks. Without any problem we are able to produce an almost infinite stream of movements in order to reach goals in the environment.

For a large part, motor behavior can be seen as problem solving. We are forced to find solutions for the problems which appear in a continuously changing environment. The obtained solutions, however, are never static, but always tailored to the actual requirements. Indeed, when the environmental constraints are never the same, the solutions can also never be the same.

This is an important point since it indicates that motor control cannot be the result of a rigid hierarchically organized system, generating efferent commands to individual muscles and joints on basis of motor programs stored in a huge neural warehouse. The control is for a large part non-hierarchical, self-organizing, and driven by multisensory input. Furthermore, the organism never functions in vacuo, disconnected from its history and without any knowledge. On the contrary, almost all actions are influenced by knowledge and experience. We have learned how to handle a cup, to ride a bicycle, to write, to play the violin, to dance. Even the most simple actions such as how to open a door are influenced by learning. We know, for example, when to push and when to pull on basis of knowledge derived from experience. Hence, motor processes continuously interact with cognitive and perceptual processes. This interaction between perception, action, and knowledge forms the basis for human motor behavior. Only in this way we are able to cope with the environmental instability [27].

I will describe the human motor system with an emphasis on flexibility and change. It will be shown that the human motor system is continuously updating itself on the basis of sensory input and activity. The above mentioned intimate relationship between perception and action, but also between cognition and action will be stressed.

The human brain is a biological system of ultimate complexity, consisting of 100 billion nerve cells (neurons), whereby each neuron is connected with thousands of other cells creating an information processing network whose detailed function is still largely unknown. For a long time it was thought that the adult brain was a fixed organ as is reflected in the famous statement of Santiago Ramon y Cajal [28]: “In adult brain centers, the nerve paths are fixed, ended, immobile. Everything may die, nothing may be regenerated”. When writing this, Cajal knew that the brain showed flexibility, but he was more or less caught in the dominant paradigm.

### Franz Joseph Gall

It was in Vienna in the early nineteenth century that Franz Joseph Gall (see [29]) presented a more optimistic view on the human brain. In a way he was far ahead of his time when he argued that brain areas could increase in size as a result of use. He claimed that the skull followed the size of the brain areas so that an increased area in the brain (reflecting a highly learned skill) could be palpated at the surface of the skull as a bulb. Gall termed his system Organologie. Later his system became known as phrenology, a term never used by Gall himself. Phrenology, fiercely defended by Johann Gaspar Spurzheim (see [29]) derailed in a series of wrong assumptions and commercial interests.

Gall’s view on the brain was unorthodox since the prevailing view of brain function at the end of the eighteenth century in Vienna was that of Albrecht von Haller (1708–1777, see [29]) who argued that the brain functioned as a whole and did not have areas where distinct faculties were localized. Gall was right about the supposed flexibility of the brain, but he looked at the wrong side. He looked at the skull and there was nothing to see, the skull does not expose what happens on the inside. After his death in 1828, Gall was slowly forgotten. He was buried in history, beside the remains of phrenology.

### The landmark experiments of Michael Merzenich

More than 200 years later in the 1980s, Merzenich and co-workers showed that neural representations (maps) of the limbs are flexible and continuously updated by our movements. Repetition of movement leads to the strengthening of these representations (enlargement), whereas inactivity or non-use results in the shrinkage of these representations. In a way we hear the echo of Gall. In a landmark experiment Merzenich et al. [30] indicated that if a body part becomes less active, such as after deafferentation, its topographical representation in the somatosensory cortex shrinks. He clearly showed that the adult human brain is not a rigid system, but a system that continuously undergoes plastic changes after alterations in the sensory flow from peripheral receptors and nerve fibers. The maps changed under the influence of input. When input was withdrawn, the maps more or less shriveled up, whereas when input was increased, the maps extended in space. Furthermore, they showed that previously existing synapses could be dramatically modified and that new synapses could be formed. Many other studies showed that central sensory representations could be reorganized, not only as a result of changes in the peripheral input in an experimental context, but also after amputation, spinal cord injury, deafferentation, after ischaemic nerve block .

In particular, the speed of these reorganization processes was impressive, which indicated that reorganization within the motor system is not an occasional state of the brain, but rather the normal ongoing condition of the human brain throughout the life span. The human motor system is reorganizing itself more or less permanently on the basis of input. In that way the motor system may differ from other systems such as the visual system. This capacity to reorganize plays a crucial role not only in learning but also in recovery of motor function after damage to the brain.

### Motor imagery

The availability of multimodal response-related input forms a crucial factor not only for the intactness of motor representations in the brain, but also for the intactness of body awareness and for learning and recovery. Against this background, learning can be seen as input-dependent plasticity that is reflected in changes in the brain.

However, since recent studies show that brain activity during the actual performance of a movement is comparable to brain activity in a task where the movement is imagined or observed instead of performed, the question becomes relevant whether for motor learning it is always necessary to actually execute the movement. In other words would it be possible to learn movements not by executing them but by imagining the movements or by observing the movements as performed by others? Does the imagination (and/or observation) of a movement result in a flow of information that is similar to the flow that is generated by the actual execution of a movement?

On the basis of research performed during the last decade, the answer on the questions is affirmative. There is ample evidence that both motor imagery and action observation indeed, play a role in (re)learning motor skills since they share a common neurophysiological basis with action execution [31–34].

It is argued that mirror neurons form a crucial factor in the explanation of the role observation and imagination play in motor learning. Mirror neurons, first identified in monkey premotor area F5, discharge when an animal performs a movement, but also when the animal observes another individual performing the same or a related movement [35, 36], for a critical discussion on mirror neurons see [37].

Motor imagery seems to rely on a network involving motor related regions including frontoparietal areas and subcortical structures, which supports the view that motor imagery and motor execution are very similar processes [38]. Motor imagery and action observation have been used in neurological rehabilitation [39], in sports [33], and in musical training [40].

Motor imagery can be described as the activation of a motor representation or motor program, while “blocking” the output mode. This activation elicits an estimation of the sensory consequences that would have taken place when the action was actually performed. A clear relation exists between motor imagery and memory. Movements stored in memory systems of the brain form the input for the sensory estimation. From clinical studies it is, indeed, known that patients with severe memory disorders show also difficulties in their ability to imagine.

### Action observation in human neonates

Human beings are excellent imitators. No other animal is more able to do so than man. Human imitation starts at a very young age. In a series of very intriguing experiments Meltzoff & Moore [41] showed that infants between 12 and 21 days of age are able to imitate both facial and manual gestures and that this behavior cannot be explained in terms of conditioning. The results implied that human neonates can equate their own unseen behaviors with gestures they see others perform. A similar study with a group of 40 infants with a mean age of 72 h (youngest 42 min) showed the same results, making it unlikely that intermodal mapping these infants displayed was learned [42].

## Conclusion

In this short paper it was attempted to show that the human motor system is a flexible non-hierarchical system, that almost literary “runs on information”. It was argued that the above cited statement of Cahal at the beginning of the twentieth century was too pessimistic, at least for the motor system. The motor system adapts and changes itself as a result of activity-driven input but also as a result of input that is generated “off-line” as is the case in motor imagery. Action observation forms a relevant additional source of sensory input.

## Part 4: learning to see beyond visual resolution by Manfred Fahle

Learning, according to Merriam-Webster [43], is the activity or process of gaining knowledge or skill by studying, practicing, being taught or experiencing something. In our case, the practicing of certain visual tasks can improve the skill of detecting and discriminating certain visual features.

There exist quite a number of different types of learning. There is first short-term learning that leads to short-term memory. Short-term learning enables us, for example, to memorize phone numbers that we hear until we are able to write them down. Another type of short-term learning involves visual impressions that we can store in short-term memory, for example when copying complex patterns.

In ophthalmology, we are more interested in the second type of learning, namely long-term learning and long-term memory. In long-term learning and memory, again, there exist two quite different types of learning. The first one deals with facts and events that can be described with words. This part of learning and memory is called explicit or declarative. The brain structure involved is mainly the medial temporal lobe; there we store facts and events from the past and learn about new facts and new events. The second type of long-term learning and memory cannot be communicated with words. It is called implicit or non-declarative learning and memory. Four subtypes of long-term learning and memory are generally discriminated. The first one is non-associative learning, namely habituation and sensitization. This is not really a long-term type of memory and learning because habituation and sensitization usually last only a few days or weeks. Habituation means that we react less strongly to a stimulus that has been presented several times in a row. On the contrary, sensitization means that we are reacting more strongly to a stimulus that was presented several times. Sensitization, of course, happens far less often than habituation. The second type of medium long-term learning and memory is called priming. It relies on the neocortex and means that a stimulus that we experienced may influence our behavior and reactions in ways that are mostly subconscious. Third, there is associative learning, namely classical and operant conditioning, as in the case of Pavlov’s dog. This type of learning and memory relies mainly on the amygdala and the cerebellum. Finally, there are procedural forms of long-term learning and memory, and personally I would count perceptual learning as one form of procedural learning, which relies on the striatum and the neocortex.

When defining perceptual learning, we can follow Gibson [44] who stated, “any relatively permanent and consistent change in the perception of a stimulus following practice or experience with this array will be considered perceptual learning.” The important points of this definition are, first, the part that perceptual learning means a relatively permanent and consistent change unlike, for example, dark adaptation. The second important point is that this improvement is the result of an active process. In the case of perceptual visual learning this improvement usually relies on training the perception and categorization of visual stimuli and often indeed very extensive training. Work by myself and others indicates that perceptual learning is not just a better use of sensory data on relatively “high” and complex levels of cortical processing, but that even early sensory and especially visual cortical areas can change their behavior as a result of training [45].

Fortunately, the processes on the cellular or neuronal level that underlie learning have been clarified by means of electrophysiological and biochemical investigations by Kandel and others [46]. Today, we can be sure that plasticity in the nervous system relies on changes at the level of synapses. Synapses can learn, for example, to set transmitter free faster, to produce more transmitter or to set free additional second messengers. Moreover, neurons may produce additional synapses to influence other neurons better. While we do not have to consider these changes here in detail, it is certainly reassuring that the underlying mechanisms of perceptual learning on the cellular level have been clarified.

**Fig. 8:**
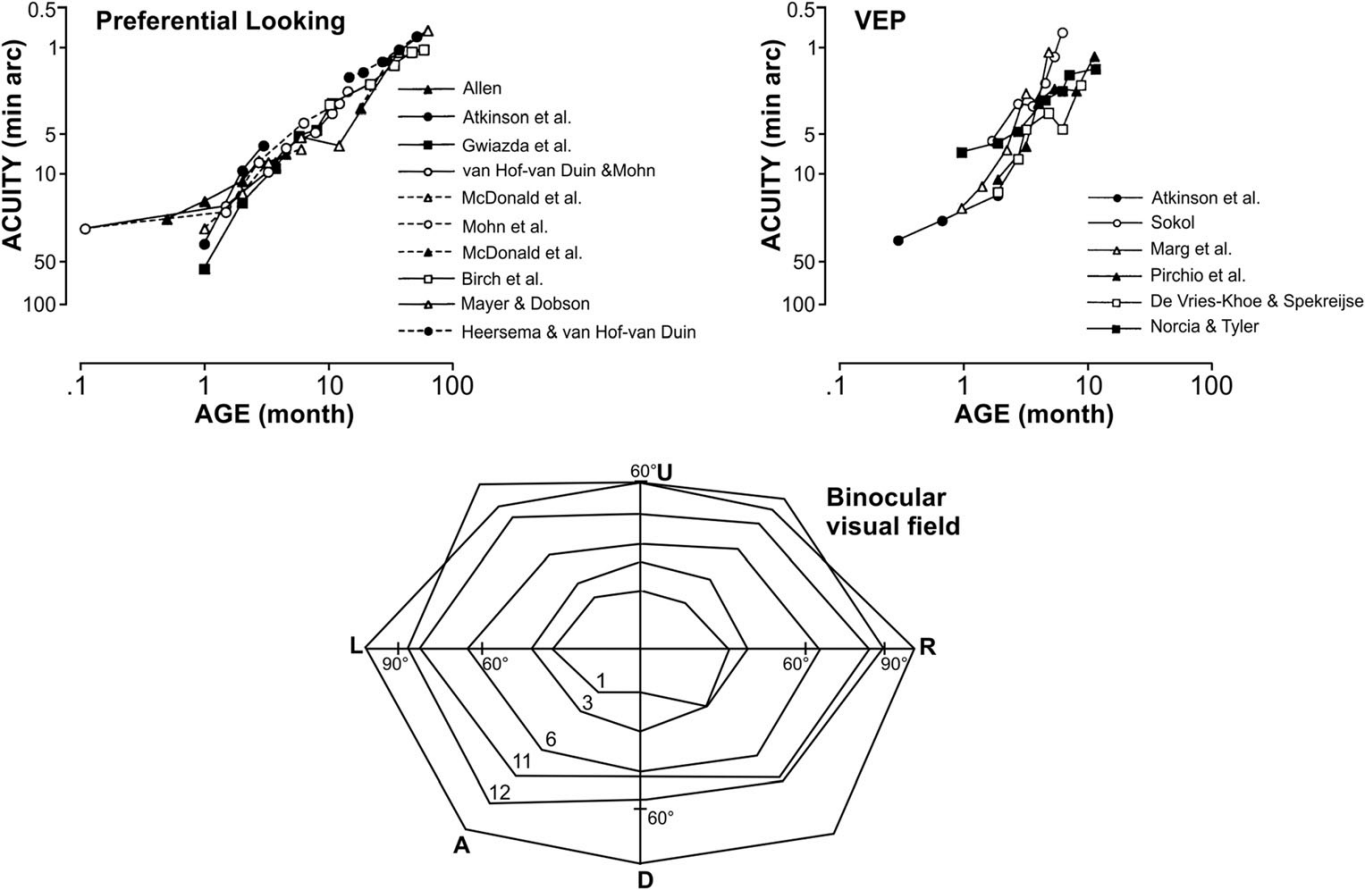
Improvement of visual acuity (preferential looking *upper left*, and VEP *upper right*), and visual field size (*bottom*; isopters shown for years) during early years of life. Results of preferential looking and visual evoked potentials from [47]

Perceptual learning is a very important process during early life. Newborns have a visual acuity clearly below 1/20 (0.05). The fast improvement of visual acuity over the first months and years of life is not only due to maturation processes, but mainly due to active learning through something I would call early perceptual learning. Both studies using visually evoked potentials (VEP) and behavioral measures, such as preferential looking, show fast improvement of visual acuity, and an increase of the visual field size[47] (see Fig. 8). Perceptual learning is not only happening during childhood, but also in adults. While most of my patients see me to get reading glasses around the age of 45 years, there are a few non-myopic ones who come up to me 10 years later. These patients insist that they are able to read or at least were able to read until recently. I tend to believe them. Perceptual learning can enable you to guess the correct letters even from rather blurred images. And there are companies that sell apps, for example for smart phones, that enable people in this age range to read without reading spectacles by learning to decipher even rather blurred letters and words.

We decided to investigate perceptual learning mainly by means of a phenomenon called visual hyperacuity. This term denotes the fact that we as humans are able to detect features that are clearly below the diameter of the photoreceptor spacing even in the foveola, for example in stereovision and when reading a Vernier scale. The features that can be resolved are in the order of magnitude of 10 arcseconds, even for unexperienced observers, and down to 2 or 3 arcseconds for very experienced observers. These low thresholds, for example when deciding whether the lower element of a Vernier target is offset to the left or to the right relative to the upper one, is really amazing when we consider that photoreceptors have a diameter of around 25 arcseconds.

When Wülfing in the nineteenth century first described these low thresholds, people concluded that the anatomists had gotten it wrong when they calculated the size of photoreceptors. At this time, the size of photoreceptors had been measured and determined to be around 25 arcseconds when converted into an angular measure. So people reasoned that photoreceptors had to be much smaller than previously thought, due to the low thresholds measured by Wülfing [48]. But the anatomists had gotten it right: photoreceptors are indeed much wider and larger than hyperacuity thresholds. Hering [49, 50] tried to resolve this paradox by postulating that the low thresholds are because Vernier stimuli extend over hundreds and thousands of photoreceptors and that the brain is able to average over these many photoreceptors. Unfortunately, this explanation was wrong as was shown by Ludvigh [51]. When three dots are presented (almost aligned), then under optimal conditions, a lateral displacement of the middle dot can be detected for deviations that are again below 10 arcseconds, i.e. clearly below the photoreceptor diameter.

Only at the end of the twentieth century, the puzzle of hyperacuity has been resolved. The underlying cause for this amazing spatial acuity lies in the fact that our optics is not at all optimal. The retinal image even of the smallest star that is a light source almost as small as a mathematical point extends on the retina over several photoreceptors. So while one photoreceptor will usually be most strongly activated, its neighbors are somewhat less strongly activated. Then, the brain is able to calculate the position of this star with a precision far below the photoreceptor diameter by comparing the relative excitations of these neighboring photoreceptors. As a consequence, the spatial resolution to pinpoint the exact position of visual features relative to each other is mainly limited by signal-to-noise ratios, rather than by photoreceptor diameter or photoreceptor distance, as long as the conditions of Shannon’s sampling theorem are fulfilled [52]. This theorem postulates that any signal can be completely reconstructed, as long as there are slightly more than two sampling points for the highest frequency that is part of this signal, in this case the image. And indeed, the density of foveal photoreceptors is sufficient to sample at least twice the highest frequency that can be produced by the optics of the eye, that is, more than 30 receptors per degree of visual angle. Hence, physics can show that there is no magic in these low perceptual thresholds in hyperacuity that enable us, for example, to detect a displacement between two lines at a distance of 100 km, once the offset is above 1.5 m!

Over the last decade we have performed quite a number of experiments on perceptual learning by using different hyperacuity tasks. Here, I will give the example of Vernier learning. As indicated above, we interpret our data as indicating that indeed to achieve the very highest performance, i.e. the very lowest thresholds, learning cannot be exclusively on relatively high levels of cortical processing but has to involve already on the early sensory cortical areas. Let me try to convince you that this hypothesis is correct. In the first experiment we presented Vernier stimuli to 12 observers. In six observers these Vernier stimuli were oriented horizontally, for the other six observers they were oriented vertically. Observers trained with these stimuli for 1 h and improved detection on average from around 50% to 70%. When we rotated the stimuli (the group that had trained with vertical stimuli now had to practice with horizontal stimuli and vice versa), the detection level dropped drastically, even slightly below 50%, and observers had to learn the new task, that only deviated from the previous task by stimulus orientation, completely from scratch, attaining 70% detection only after about one additional hour of training. In a control group where we did not change stimulus orientation no such drop of performance occurred.

We then repeated the experiment in a lengthier version, training observers for 5 h on five consecutive days. The thresholds improved from around 13 arcseconds to about slightly below 10 arcseconds during that time. Then, again, we changed orientation by 90 degrees so that observers who had trained with vertical Verniers now had to respond to horizontal Verniers. Thresholds increased strongly, to above 15 arcseconds, that is, even worse than in the untrained observers. This is to say that surprisingly, extensive training with one stimulus orientation improved performance for this orientation, but decreased performance for the stimuli rotated by 90 degrees. Again, performance improved over five additional hours of additional training to achieve the level attained for the first orientation only after these 5 h of training. This is to say that perceptual learning in the hyperacuity range is highly specific for stimulus orientation.

In a second experiment, we trained observers with one eye patched. Six observers started with the left eye patched while six further observers started with the right eye patched. The improvement was similar as in the experiment with stimulus rotation. After 1 h of training, the drop of performance after changing patch side was less pronounced than for the rotation of stimulus orientation, but for the companion experiment with long-term learning of 5 h per observer, we again found a strong decrease of performance when observers switched from seeing with one eye to the partner eye (even slightly) below the level of untrained observers. Please note that for all of these experiments, new observers were recruited for each new experiment.

These results and additional results we obtained, for example, by using visual evoked potentials that showed significant change as a result of perceptual learning already over the occipital pole [53], lead us to the conclusion already mentioned above, that perceptual learning can change processing already on a very early level of cortical computation before the the inputs from the two eyes are combined. If perceptual learning improved performance through better evaluation of sensory signals on higher levels of cortical processing, one would have to expect that improvements generalize from one eye to the other. One has to keep in mind that, due to tremor and small eye movements, stimuli will fall on different parts of the retina over the course of the experiment. Different parts of the same retina will differ from each other as much or maybe even more than corresponding parts of both eyes. Hence, an improvement that is specific for one eye strongly suggests that this improvement is mediated on very early levels of visual information processing that are still monocularly activated. This is to say that the old view of a hard-wired early visual cortex, as proposed for example by Marr and colleagues [54], does no longer hold true. Quite to the contrary, the early sensory cortical areas seem to keep some plasticity even in adults.

This has consequences not only for the therapy of amblyopia, but also for stroke patients. As long as signals reach the visual cortex, learning and compensatory mechanisms are able to improve perception and discrimination of objects. The essential condition to keep in mind is that signals from the retina have to arrive at the brain. Phenomena such as blind-sight seem to indicate that these signals do not necessarily have to arrive in the primary visual cortex, but other parts of the cortex may also be able to subserve some type of rudimentary vision. If, on the other hand, fibers are destroyed, as is the case in glaucoma or strokes on the level of the thalamus, then the resulting visual field defects cannot be made to disappear by means of perceptual learning. Training can improve the way that the visual cortex analyzes and categorizes visual stimuli, but can never compensate absolute visual field defects caused by lesions on very early levels of the visual system.

To conclude, we find that there are a number of different forms of learning and have reminded ourselves that learning dramatically improves seeing in infants and can improve visual perception at least slightly in patients and in presbyopes. We also find that in several so-called hyperacuity tasks, such as Vernier acuity and stereopsis, observers achieve spatial resolution far below the photoreceptor diameter and photoreceptor spacing even in the foveola and can thus, at least after extensive training, attain thresholds that are far below photoreceptor diameters. But improvement in perceptual learning seems under most conditions to be very specific for the exact task trained and therefore indicative of changes that involve even the level of early sensory cortical areas. Extensive research is presently under way to find training procedures leading to perceptual learning that generalizes to new tasks.
